# Extracellular Environment-Controlled Angiogenesis, and Potential Application for Peripheral Nerve Regeneration

**DOI:** 10.3390/ijms222011169

**Published:** 2021-10-16

**Authors:** Shingo Saio, Kanna Konishi, Hirofumi Hohjoh, Yuki Tamura, Teruaki Masutani, Arunasiri Iddamalgoda, Masamitsu Ichihashi, Hiroshi Hasegawa, Ken-ichi Mizutani

**Affiliations:** 1 Laboratory of Stem Cell Biology, Graduate School of Pharmaceutical Sciences, Kobe Gakuin University, 1-1-3 Minatojima, Chuo-ku, Kobe 650-8586, Japan; prirt136@s.kobegakuin.ac.jp (S.S.); ptuzm172@s.kobegakuin.ac.jp (K.K.); tamura27@pharm.kobegakuin.ac.jp (Y.T.); mm_ichihashi@syuhakukai.org.jp (M.I.); 2 Laboratory of Hygienic Sciences, Kobe Pharmaceutical University, 4-19-1, Motoyamakitamachi, Higashinada-ku, Kobe 658-8558, Japan; h-hohjoh@kobepharma-u.ac.jp; 3 Research & Development Dept., Ichimaru Pharcos Co., Ltd., 318-1 Asagi, Motosu 501-0475, Japan; masutani-teruaki@ichimaru.co.jp (T.M.); arunasiri@ichimaru.co.jp (A.I.); 4 Medical Education Development Center, Gifu University School of Medicine, 1-1 Yanagido, Gifu 501-1194, Japan

**Keywords:** angiogenesis, extracellular matrix, proteoglycan, peripheral nerve regeneration

## Abstract

Endothelial cells acquire different phenotypes to establish functional vascular networks. Vascular endothelial growth factor (VEGF) signaling induces endothelial proliferation, migration, and survival to regulate vascular development, which leads to the construction of a vascular plexuses with a regular morphology. The spatiotemporal localization of angiogenic factors and the extracellular matrix play fundamental roles in ensuring the proper regulation of angiogenesis. This review article highlights how and what kinds of extracellular environmental molecules regulate angiogenesis. Close interactions between the vascular and neural systems involve shared molecular mechanisms to coordinate developmental and regenerative processes. This review article focuses on current knowledge about the roles of angiogenesis in peripheral nerve regeneration and the latest therapeutic strategies for the treatment of peripheral nerve injury.

## 1. Angiogenesis and Vascular Endothelial Growth Factor Signaling

Blood vessels, including arteries, veins, and capillaries, are densely spread throughout the body. It takes approximately 1 min to complete 1 round of systemic blood circulation through these blood vessels. The circulated blood transports oxygen, nutrients, and fluid to cells throughout the body. Capillaries have a relatively simple structure consisting of endothelial cells that cover the inner layer and pericytes surrounding the outer layer. Capillaries are also responsible for filtering toxic byproducts, thus providing an appropriate vascular environment for the maintenance of cell functions [1]. However, the structure of the vascular network is extremely diverse, and blood vessels create tissue-specific microenvironments. Anatomical findings show great vascular diversity, but the molecular mechanism of how such tissue-specific capillary milieus are generated has remained unclear. Therefore, the orchestration of the functional blood vessel network requires coordinated signaling among adjacent cells and microenvironmental factors, leading to hierarchically branched circuitry. The process of angiogenesis encompasses an entire set of events that leads to the development of new tubular structures, and subsequent association with pericytes or smooth muscle cells, from assembling activated endothelial cells, including sprouting, proliferation, migration, lumen formation, and the dynamic regulation of cell-cell contacts within endothelial cells [2].

**Figure 1:**
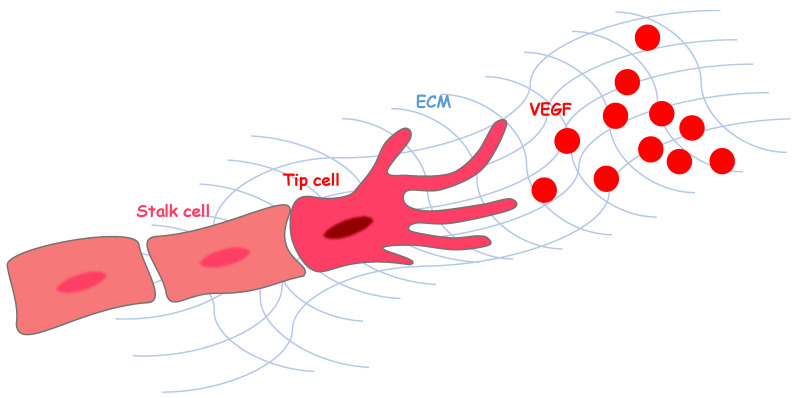
Extracellular environment-guided vascular endothelial growth factor (VEGF) gradients regulate the differentiation of endothelial cells, leading to the construction of vascular plexuses with regular, tissue-specific morphology. The spatiotemporal localization of angiogenic signaling molecules, such as VEGF in the extracellular matrix (ECM), directs angiogenesis, thus the proliferating endothelial stalk cells are led by endothelial tip cells that extend and retract filopodia to control migration and elongation.

The principal angiogenic growth factors, such as vascular endothelial growth factor, basic fibroblast growth factor family (bFGF), insulin-like growth factor (IGF), hepatocyte growth factor (HGF), platelet-derived growth factor (PDGF), and transforming growth factor-β (TGF-β), have common characteristics, enabling them to bind with the extracellular matrix (ECM), which is required for their cellular behavior to direct angiogenesis toward the hypoxic areas (Figure 1) [3]. Among these growth factors, VEGF is the master regulator of both physiological and pathological angiogenesis, and it is capable, when delivered as a single factor, of starting the complex cascade of events leading to new vascular growth [4]. VEGF is, therefore, a major potential therapeutic target [5].

The mammalian VEGF family members include VEGF-A, B, C, D, E, and placental growth factor. These proteins have different binding affinities for three tyrosine kinase receptors: VEGF receptor 1 (VEGFR1, Flt1), VEGFR2 (KDR/Flk-1), and VEGFR3 (Flt4) [6,7]. Multiple major angiogenic processes are generated by signaling involving VEGF-A and VEGFR2. The kinase activity of VEGFR1 in response to VEGF is much weaker than that of VEGFR2, and it functions as a negative regulator of VEGFR2, leading to regulation of the assembly of endothelial cells, which is important for the maintenance of functional vessels [7]. Thus, VEGF signaling drives physiological angiogenesis via a balance between VEGFR1 and VEGFR2. The establishment of a ramified pattern in the tissues requires the functional differentiation of endothelial cells into “tip” and “stalk” cells in response to microenvironmental VEGF gradients (Figure 1) [8]. Growing vessels, activated by VEGF, become endothelial tip cells, which have a high expression of VEGFR2. These cells extend thin filopodial processes and migrate toward the source of VEGF (Figure 1). Endothelial stalk cells proliferate behind the guiding tip cells and extend angiogenic sprouts to support the formation of the new vessel [8]. Recent studies have shown that the tip and stalk phenotypes are not permanent once acquired, but they are dynamically switched throughout angiogenesis [9,10]. The presence of an adequate number of tip and stalk cells during sprouting, together with a regulated balance between stalk cell proliferation and tip cell migration, are essential for the construction of tissue-specific vascular networks with appropriate levels of branching complexity. Recently, we have found that highly vascularized and avascular regions are precisely regulated in the developing neocortex tissue, and capillary vessels form stereographically regular three-dimensional patterns [11]. Each capillary network shows distinct vascular morphology in the ventricular zone (VZ) and cortical plate (CP). The honeycomb-patterned vessels consist of endothelial cells predominantly expressing VEGFR2 in the VZ, and the vertically-oriented vessels consist of endothelial cells predominantly expressing VEGFR1 in the CP [11]. Moreover, we found an avascular region, without capillary vessel invasion, specifically constructed in the ventricular surface where mitotic progenitors are located, and neural progenitors in the VZ showed high VEGF expression transiently, thereby, attracting vascular endothelial tip cells with high VEGFR2 expression. Although the morphological differences between each vascular plexus, with different VEGF receptor expression levels, have been investigated, the functional characteristics of the endothelial cells that distinguish region-specific properties are still poorly understood. We found that the expression of several ECM components is markedly different between the different endothelial cell types (data not shown).

## 2. Angiogenesis and Extracellular Environments

**Figure 2:**
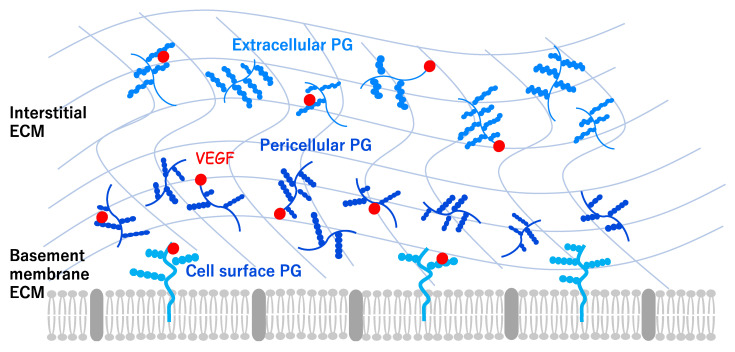
Transmembrane, pericellular, and extracellular proteoglycan (PG) family proteins regulate various cellular functions through cell-cell and cell-extracellular matrix (ECM) interactions. The vascular ECM plays a fundamental role as a reservoir for growth factors such as vascular endothelial growth factor (VEGF).

The ECM is known to contribute to the angiogenesis in multiple ways [12]. The spatiotemporal regulation of angiogenic signals in the ECM plays a critical role in tissue vascularization [13]. The vascular ECM is composed of a basement membrane (BM) and an interstitial ECM (Figure 2), and this cellular microenvironment is crucial to the formation of complex three-dimensional vascular networks. The composition and structure of blood vessels differ in their lumen size and physiological functions, depending on the surrounding environment [14,15]. For example, capillary vessels lack smooth muscle cells and the associated interstitial ECM, and consist of a single endothelial cell layer, the underlying BM, and surrounding pericytes. The large vessels consist of endothelial cells, a surrounding smooth muscle cell layer, and the interstitial ECM components, which provide stiffness and mechanical integrity. The core components of the vascular ECM are laminin 411 and 511, collagen IV, XVIII, VI, VIII, fibronectin, and proteoglycans (PGs), such as Aggrecan and Perlecan. The ECM is a crucial regulator of angiogenesis and vascular homeostasis [16]. The vascular ECM also plays a role as a reservoir for growth factors that influence angiogenesis via signaling pathways.

ECM-bound VEGF elicits a prolonged activation of VEGFR2 and stimulates angiogenesis, mediated by Integrin signaling, indicating the existence of specific components within the ECM that sustain angiogenesis [17]. It has recently become apparent that PGs are key regulators of angiogenesis in the brain (Tamura et al., unpublished).

PGs are an extremely diverse group of core proteins, with one or more glycosaminoglycan (GAG) side chains covalently attached [18]. Cell surface, pericellular, and extracellular PGs play an important role in cell-cell and cell-ECM interactions and signaling (Figure 2), and they influence diverse cellular processes such as cell proliferation and differentiation, as well as tissue repair [19,20,21]. Various PGs, with several different types of core proteins and GAGs, are expressed in each cell subtype and tissue. For example, the chondroitin sulfate PG (CSPG) aggrecan, heavily glycosylated by CS and keratan sulfate (KS) chains, is abundantly present not only in cartilage and the intervertebral discs but also in other tissues, including the brain and blood vessels. In the developing chick embryo, the expression of aggrecan is mostly localized to the outer region of the aorta [18,22]. Aggrecan has been associated with cardiovascular disease, with increased or decreased aggrecan reported in aortic aneurysms, atherosclerosis, venous hypertension, and cardiac valve anomalies [22]. A recent study reported that aggrecan has been identified in human normal aorta by the proteoglycanome analysis, but massive aggrecan accumulation and reduced expression of proteoglycanase genes, such as ADAMTS5, was confirmed in thoracic aortic aneurysm and dissection [23]. Another CSPG, versican, is also expressed in several tissues, including the vasculature, especially vascular smooth muscle cells. In the aorta of the developing chick embryo, high levels of expression of versican have been detected throughout the aortic wall [24]. Perlecan, a secreted heparan sulfate PG (HSPG), is one of the major components of the endothelial BM, and associates with VEGF-A via its HS side chains to regulate vascularization and tissue development [25]. Knockdown of perlecan expression in the zebrafish embryo has been shown to be necessary for the turnover and proper localization of VEGF-A [26]. Perlecan has also been involved in correct vascular patterning because perlecan activates the VEGF-A-induced phosphorylation of VEGFR2 in endothelial cells [27]. The perlecan-null mouse exhibited vessel modeling defects in the developmental pattern of the coronary artery [28], but it did not exhibit any major defects in developmental angiogenesis, probably due to the effects of compensatory mechanisms. The perlecan C-terminal domain V also acts as a pro-angiogenic factor by enhancing the expression of VEGF in brain endothelial cells [29,30]. Recent reports have indicated that the perlecan C-terminal domain V regulates pericyte recruitment by the activation of PDGF receptor β, and it repairs the blood-brain-barrier (BBB) after ischemic stroke [31]. Agrin is another HSPG secreted in the vascular BM. Agrin is considered to be a marker of tumor angiogenesis because it recruits endothelial cells within tumors. A recent study has shown that Agrin regulates VEGFR2 stability to sustain angiogenesis and adherence between endothelial cells and cancer cells [32]. Agrin might also contribute to the stabilization of the adherens junction proteins, such as β-catenin and VE-cadherin, in brain microvascular endothelial cells, leading to maintenance of the BBB [33]. Overall, these findings suggest that the vascular ECM is a crucial regulator not only of the vascular cells but also of the interactions between endothelial cells and surrounding cells.

## 3. Angiogenesis in Peripheral Axon Regeneration

During embryogenesis, the development of the vascular and nervous systems is guided by similar signaling pathways [34,35]. Close interactions between vascular and neural networks are necessary for physiological homeostasis such as the glymphatic system and interstitial fluid system [1,36,37]. Moreover, the functional degradation of nervous system upon aging, injury, and various diseases is caused by mutual interactions among multiple cell types, including neurons, glial cells, immune cells, and vascular cells [1,35,36,37]. Targeting of the vascular system to promote neural regeneration following injury is, therefore, an emerging therapeutic approach. However, a limitation of this strategy is that the molecular interactions between emergent angiogenesis and neural differentiation during regeneration are poorly understood. In this section, we review the role of angiogenesis in peripheral axon regeneration, as an experimental model, and discuss the therapeutic application of neurovascular regeneration.

The neurons in the peripheral nervous system can regenerate their axons following complete nerve transection. Although this capacity cures injured nerves in humans, the rate of spontaneous axon regeneration is not high enough to completely heal damaged nervous tissues. To support spontaneous regeneration in damaged peripheral nerves, it is important to understand the molecular mechanisms of peripheral axon regeneration after injury. 

**Figure 3:**
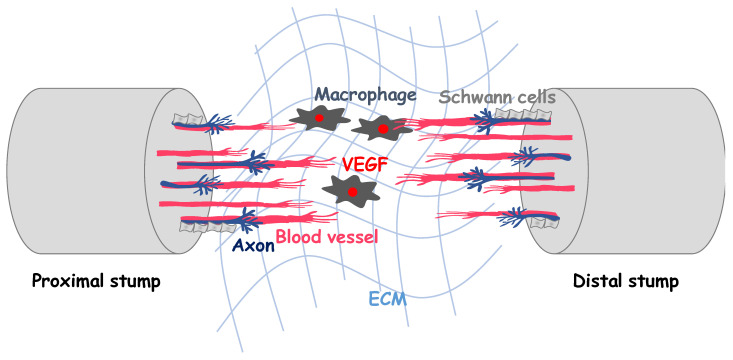
Macrophage-derived vascular endothelial growth factor (VEGF)-A facilitates angiogenesis and Schwann cell migration, along with guiding newly formed blood vessels, leading to peripheral nerve regeneration. Therefore, tissue-engineered conduits with vascular extracellular matrix (ECM) may improve the outcomes of peripheral nerve regeneration.

After complete nerve transection, proximal and distal nerve stumps retract, and a gap is generated between the stumps (Figure 3). This gap is filled with new tissue, called the nerve bridge, which connects the nerve stumps by an unknown mechanism. The nerve bridge recruits macrophages, which produce VEGF-A and activate angiogenesis in the nerve bridge (Figure 3) [38,39]. In both the proximal and the distal stumps, Schwann cells are de-differentiated and migrate into the nerve bridge to form discrete cell cords, known as Schwann cell cords, which later guide the regenerating axons [40]. De-differentiated Schwann cells in the cords secrete ECM proteins and nerve growth factors to support axon growth. The axons regenerating from the proximal stump grow past the nerve bridge and travel into the distal stump. Finally, the regenerated axons are myelinated with re-differentiated Schwann cells. It has long been known that angiogenesis occurs in the nerve bridge during peripheral nerve regeneration. However, the functional role that angiogenesis plays in axon regeneration is unclear.

After peripheral nerve injury, the number of endoneurial capillaries in the injured nerve is significantly increased, and the number of newly formed capillaries is correlated with the extent of axon regeneration [41]. These observations suggest that angiogenesis could have a supportive function in axon regeneration. Recently, Cattin et al. indicated that the newly formed blood vessels provide “tracks” for Schwann cell migration in the nerve bridge [39]. They found that angiogenesis in the nerve bridge is induced by macrophage-derived VEGF-A (Figure 3) and precedes the migration of Schwann cells into the nerve bridge. The migration of Schwann cells is facilitated and guided by newly formed blood vessels, which function as a “guiding structure” for Schwann cell migration (Figure 3). Blood vessel endothelial cells express and secrete various signaling molecules [42,43]. However, it remains unknown which endothelial cell-derived signaling molecules are important for axon regeneration. Therefore, to identify those molecules, we examined the change in expression of secreted factors in injured mouse sciatic nerves induced by cediranib, a VEGFR2 inhibitor. Cediranib attenuated the nerve injury-induced upregulation of some secreted factors, such as IGF-1, Neurotrophin-3, PDGF-β, interleukin (IL)-6, and TGF-β1 (Hohjoh et al., unpublished). These factors are known to be induced in vascular endothelial cells after peripheral nerve injury [44]. The functional and molecular roles of these secreted factors are currently under investigation.

If the gap between the proximal and distal stumps after nerve transection is too wide, a nerve bridge cannot form, and axon regeneration does not succeed. In such cases, transplantation of autologous nerve tissue, or implantation of an artificial conduit connecting both stumps, can lead to effective axon regeneration. As in spontaneous recovery, angiogenesis occurs in the transplant and conduit and supports the regeneration of nerve tissue. Inhibition of angiogenesis prevents invasion of Schwann cells into the conduit and axon regeneration [45,46,47], while implantation of a vascular nerve conduit, or cultured human umbilical vein endothelial cells with a tube-like structure in collagen hydrogel, promotes peripheral nerve regeneration [48,49].

In addition to their function as a guiding structure for Schwann cells and an angiocrine source of secreted factors, the new blood vessels in the conduit support axon regeneration by regulating T lymphocytes. Blood vessel endothelial cells in the conduit induce the accumulation of T lymphocytes, which, in turn, recruit eosinophils [50]. Eosinophils secrete IL-4, which could be essential for neuronal survival, axon regeneration, and the remyelination of regenerated axons [50,51]. Little is known about the significance of T lymphocytes and eosinophils in the spontaneous recovery process. In our recent study, T lymphocytes were recruited to the nerve bridge in a mouse sciatic nerve transection model. The inhibition of T lymphocyte accumulation in the nerve bridge, by FTY720, which blocks T lymphocyte egress from the lymph nodes, prevented the recruitment of Schwann cells and axon regeneration, but not angiogenesis (Hohjoh et al., unpublished). Thus, T lymphocyte accumulation, induced by newly formed blood vessels, might also play an important role in spontaneous repair.

## 4. Vascularization Strategies for Peripheral Nerve Regeneration

Angiogenesis is a potential target of treatment for peripheral nerve repair. Some strategies for controlling angiogenesis have been reported to be effective in animal models. The best known is VEGF-A, which is the most potent angiogenic factor, and it is required for the growth of peripheral axons [52,53]. The implantation of a conduit supplemented with VEGF-A induces angiogenesis, Schwann cell invasion, and axon regeneration [54]. This approach facilitates the recovery of electrophysiological and motor functions [55,56]. Alternatively, conduits vascularized with blood vessels, or those made with a vein segment, are also effective for targeting this injury [57,58]. These recent advances indicate that artificial control of angiogenesis will be a useful technique for the clinical treatment of peripheral nerve injury and diseases, although there have been some controversial results [59]. Hence, it is important to identify the specific ECM components and angiogenic factors required for efficient vascularization in nerve regeneration.

For VEGF-A signaling to function efficiently in vascularization strategies, the extracellular microenvironment is also important. The extracellular microenvironment is not only a reservoir of growth factors and calcium [60,61], but it also plays a role in tissue-specific stiffness and mechanotransduction, which greatly contributes to the regulation of transplanted cells and ligands [62,63]. Moreover, it has been reported that mechanotransduction through the extracellular microenvironment activates signals such as migration, proliferation, apoptosis, and differentiation in cells [62]. In other words, an approach that includes specific stiffness of each tissue by the optimization of extracellular microenvironment, as well as the application of ligands and cells, is important in order to respond to the complex biological system.

PGs, components of the ECM, contribute to the maintenance of function and high activity of VEGF-A during angiogenesis. PGs not only contribute to the stiffness of the tissues [18], but they are also known to be factors involved in the activity of receptor tyrosine kinase [61,64]. We have previously reported that chondroitin sulfate PGs enhance the activity of several receptor tyrosine kinase, and this effect can be expected in angiogenesis [21]. Furthermore, the effect of GAGs, alone, on cell proliferation was significantly weakened compared to that effect of PGs, with GAGs bound to the core protein, suggesting that the signal between intact and metabolized PGs may be different [21]. When using factors as biomaterials that constitute the extracellular microenvironment, it is important to select and combine factors that are optimal for vascularization strategies.

Therefore, tissue-engineered conduits, such as biomaterials with vascular ECM, should be developed and may improve the outcomes of peripheral nerve regeneration, especially for the successful repair of large and long segmental defects.

## 5. Concluding Remarks and Perspectives

The reconstruction of the blood vessel plays a key role in regenerative medicine because angiogenesis is required not only for the restoration of the blood supply to ischemic tissues but also for triggering the differentiation of surrounding cells. VEGF has long been considered to be a major regulator of angiogenesis under both physiological and pathological conditions. However, angiogenesis is a complex process, and the way in which the localization, concentration, and bioavailability of angiogenic factors are regulated is far from understood. Future studies are needed to develop therapeutic angiogenesis approaches for peripheral axon regeneration, utilizing specific vascular ECM.

## Data Availability

Not applicable.
